# Supplementary material for: Multiplex Assays in Allergy Diagnosis: Allergy Explorer 2 versus ImmunoCAP ISAC E112i
Source: Diagnostics (Basel). 2024 May 8;14(10):976. doi: 10.3390/diagnostics14100976 (PMC11119049; doi:10.3390/diagnostics14100976)
Supplement: Supplementary file 1 [file diagnostics-14-00976-s001.zip › Figure_S1.pdf]

a. Comparison of ALEX^{2} extracts with corresponding ALEX^{2} components

ALEX^{2} extract positive and corresponding ALEX^{2} component negative	ALEX^{2} extract positive and corresponding ALEX^{2} component positive	ALEX^{2} extract negative and corresponding ALEX^{2} component positive
66.7% (8)	Amb a / Amb a 1: 25.0% (3)	8.3% (1)
46.2% (6)	Amb a / Amb a 4: 3.8% (5)	15.4% (2)
21.4% (3)	**Amb a / Amb a 1 and 4**: 57.1% (8)	21.4% (3)
80.0% (8)	Ana o / Ana o 2: 20.0% (2)	0
36.4% (4)	Ana o / Ana o 3: 54.5% (6)	9.1% (1)
25.0% (3)	**Ana o / Ana o 2 and 3**: 66.7% (8)	8.3% (1)
39.5% (15)	Api m / Api m 1: 55.3% (21)	5.3% (2)
25.0% (10)	Api m / Api m 10: 65.0% (26)	10.0% (4)
4.8% (2)	**Api m / Api m 1 and 10**: 81.0% (34)	14.3% (6)
15.6% (5)	Art v / Art v 1: 62.5% (20)	21.9% (7)
71.0% (22)	Art v / Art v 3: 9.7% (3)	19.4% (6)
10.5% (4)	**Art v / Art v 1 and 3**: 55.3% (21)	34.2% (13)
71.4% (5)	**Ber e / Ber e 1**: 28.6% (2)	0
0	**Bos d meat / Bos d 6**: 25.0% (1)	75.0% (3)
100% (2)	Bos d milk / Bos d 4: 0	0
100% (2)	Bos d milk / Bos d 5: 0	0
0	Bos d milk / Bos d 8: 100% (2)	0
0	**Bos d milk / Bos d 4,5 and 8**: 100% (2)	0
33.3% (1)	**Can s / Can s 3**: 33.3% (1)	33.3% (1)
100% (3)	**Che a / Che a 1**: 0	0
0	**Cla h / Cla h 8**: 33.3% (1)	66.6% (2)
50.0% (2)	**Clu h / Clu h 1**: 50.0% (2)	0
4.2% (4)	**Cor a pollen / Cor a 1.0103**: 75.8% (72)	20.0% (19)
0	**Cyn d / Cyn d 1**: 86.1% (130)	13.9% (21)
0	**Dau c / Dau c 1**: 96.8% (30)	3.2% (1)
75.0% (6)	**Fag e / Fag e 2**: 25.0% (2)	0
11.6% (10)	**Fra e / Fra e 1**: 88.4% (76)	0
50.0% (1)	Gad m / Gad m 1: 50.0% (1)	0
50.0% (1)	Gad m / Gad m 2 and 3: 50.0% (1)	0
0	**Gad m / Gad m 1,2 and 3**: 100% (2)	0
72.7% (8)	Gal d white / Gal d 1: 18.2% (2)	9.1% (1)
40.0% (4)	Gal d white / Gal d 2: 60.0% (6)	0
60.0% (6)	Gal d white / Gal d 3: 40.0% (4)	0
54.4% (6)	Gal d white / Gal d 4: 36.4% (4)	9.1% (1)
90.9% (10)	Gal d white / Gal d 5: 0	9.1% (1)
23.1% (3)	**Gal d white / Gal d 1,2,3,4 and 5**: 53.8% (7)	23.1% (3)
28.6% (2)	**Mac inte / Mac i 2/S Albumin**: 71.4% (5)	0
77.8% (7)	**Pap s / Pap s 2/S Albumin**: 22.2% (2)	0
9.1% (1)	**Par j / Par j 2**: 72.7% (8)	18.2% (2)
33.3% (2)	**Per a / Per a 7**: 16.7% (1)	50.0% (3)
85.7% (6)	**Pla l / Pla l 1**: 14.3% (1)	0
8.3% (1)	**Pol d / Pol d 5**: 66.7% (8)	25.0% (3)
50.0% (1)	**Raj c / Raj c Parvalbumin**: 50.0% (1)	0
94.7% (18)	**Sal k / Sal k 1**: 0	5.3% (1)
0	**Sal s / Sal s 1**: 50.0% (1)	50.0% (1)
0	**Sco s / Sco s 1**: 50.0% (1)	50.0% (1)
41.7% (5)	**Ses i / Ses i 1**: 41.7% (5)	16.7% (2)
50.0% (2)	**Sin / Sin a 1**: 50.0% (2)	0
77.8% (7)	**Sola l / Sola l 6**: 0	22.2% (2)
11.1% (1)	**Sus d meat / Sus d 1**: 11.1% (1)	77.8% (7)
33.3% (1)	**Thu a / Thu a 1**: 33.3% (1)	33.3% (1)
30.0% (3)	**Tyr p / Tyr p 2**: 20.0% (2)	5 50.0% (5)
83.3% (15)	Ves v / Ves v 1: 16.7% (3)	0
2.9% (1)	Ves v / Ves v 5: 48.6% (17)	48.6% (17)
2.9% (1)	**Ves v / Ves v 1 and 5**: 48.6% (17)	48.6% (17)
0	**Zea m / Zea m 14**: 66.7% (6)	33.3% (3)

b. Comparison of ALEX^{2} extracts with corresponding ISAC components

ALEX^{2} extract positive and corresponding ISAC component negative	ALEX^{2} extract positive and corresponding ISAC component positive	ALEX^{2} extract negative and corresponding ISAC component positive
66.7% (8)	**Amb a / Amb a 1**: 25.0% (3)	8.3% (1)
4.8% (2)	Art v / Art v 1: 54.8% (23)	40.5% (17)
75.9% (22)	Art v / Art v 3: 10.3% (3)	13.8% (4)
2.2% (1)	**Art v / Art v 1 and 3**: 52.2% (24)	45.7% (21)
71.4% (5)	**Ber e / Ber e 1**: 28.6% (2)	0
0	**Bos d meat / Bos d 6**: 33.3% (1)	66.7 (2)
66.7% (2)	Bos d milk / Bos d 4: 0	33.3% (1)
66.7% (2)	Bos d milk / Bos d 5: 0	33.3% (1)
50.0% (1)	Bos d milk / Bos d 8: 50.0% (1)	0
25.0% (1)	**Bos d milk / Bos d 4,5 and 8**: 25.0% (1)	50.0% (2)
12.5% (2)	**Che a / Che a 1**: 6.3% (1)	81.3% (13)
0	**Cla h / Cla h 8**: 50.0% (1)	50.0% (1)
11.6% (10)	**Cor a pollen / Cor a 1.0101**: 76.7% (66)	11.6% (10)
4.5% (7)	**Cyn d / Cyn d 1**: 78.8% (123)	16.7% (26)
100% (8)	**Fag e / Fag e 2**: 0	0
50.0% (1)	Gad m / Gad c 1: 50.0% (1)	0
60.0% (6)	Gal d white / Gal d 1: 40.0% (4)	0
80.0% (8)	Gal d white / Gal d 2: 20.0 (2)	0
77.8% (7)	Gal d white / Gal d 3: 11.1% (1)	11.1% (1)
100% (10)	Gal d white / Gal d 4: 0	0
100% (10)	Gal d white / Gal d 5: 0	0
27.3% (3)	**Gal d white / Gal d 1,2,3,4 and 5**: 63.6% (7)	9.1% (1)
9.1 (1)	**Par j / Par j 2**: 72.7% (8)	18.2% (2)
75.0% (18)	**Sal k / Sal k 1**: 8.3% (2)	16.7% (4)
90.0% (9)	**Ses i / Ses i 1**: 10.0% (1)	0
