# Supplementary material for: Multiplex Assays in Allergy Diagnosis: Allergy Explorer 2 versus ImmunoCAP ISAC E112i
Source: Diagnostics (Basel). 2024 May 8;14(10):976. doi: 10.3390/diagnostics14100976 (PMC11119049; doi:10.3390/diagnostics14100976)

CCD INHIBITION in ALEX^2

MUXF3 + [n]	MUXF3 + [%]	MUXF3 + and Hom s LF + [n]	MUXF3 + and Hom s LF + [%]	MUXF3 + and Hom s LF - [n]	MUXF3 + and Hom s LF - [%]
52/216	24.1	6/52	11.5	46/52	88.5

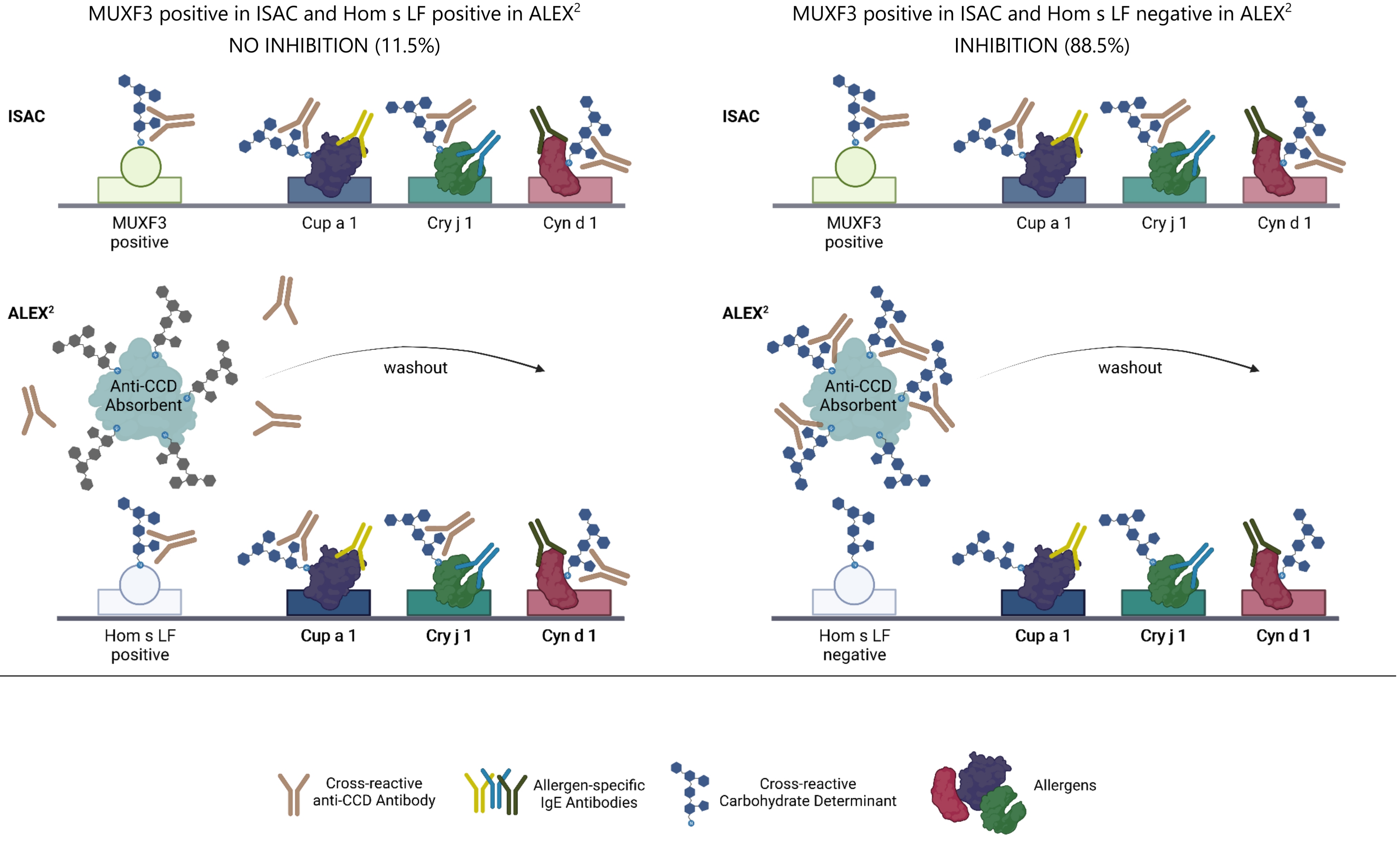

Supplement: Supplementary file 1 [file diagnostics-14-00976-s001.zip › Figure_S2.pdf]
