# Supplementary material for: Multiplex Assays in Allergy Diagnosis: Allergy Explorer 2 versus ImmunoCAP ISAC E112i
Source: Diagnostics (Basel). 2024 May 8;14(10):976. doi: 10.3390/diagnostics14100976 (PMC11119049; doi:10.3390/diagnostics14100976)
Supplement: Supplementary file 1 [file diagnostics-14-00976-s001.zip › Table_S1.pdf]

Table S1. Correlation between Sensitization, Allergy Symptoms, and Pollen/Spore Calendar. Demographic and Clinical Data of Patients with Allergies.

Patients with any sensitization to	Match of allergy symptoms with pollen/spore calendar when ALEX[2] was used to analyze sensitization [%]	Match of allergy symptoms with pollen/spore calendar when ISAC was used to analyze sensitization [%]
Alternaria*	81.8	80.0
Grass pollen	76.7	77.9
Tree pollen	50.6	55.0
Weed pollen	41.0	38.4

*For all the Alternaria sensitizations, co-sensitization with pollen allergens was present.
